# Subcellular location prediction of proteins using support vector machines with alignment of block sequences utilizing amino acid composition

**DOI:** 10.1186/1471-2105-8-466

**Published:** 2007-11-30

**Authors:** Takeyuki Tamura, Tatsuya Akutsu

**Affiliations:** 1 Bioinformatics Center, Institute for Chemical Research, Kyoto University, Gokasho, Uji, Kyoto 611-0011, Japan

## Abstract

**Background:**

Subcellular location prediction of proteins is an important and well-studied problem in bioinformatics. This is a problem of predicting which part in a cell a given protein is transported to, where an amino acid sequence of the protein is given as an input. This problem is becoming more important since information on subcellular location is helpful for annotation of proteins and genes and the number of complete genomes is rapidly increasing. Since existing predictors are based on various heuristics, it is important to develop a simple method with high prediction accuracies.

**Results:**

In this paper, we propose a novel and general predicting method by combining techniques for sequence alignment and feature vectors based on amino acid composition. We implemented this method with support vector machines on plant data sets extracted from the TargetP database. Through fivefold cross validation tests, the obtained overall accuracies and average MCC were 0.9096 and 0.8655 respectively. We also applied our method to other datasets including that of WoLF PSORT.

**Conclusion:**

Although there is a predictor which uses the information of gene ontology and yields higher accuracy than ours, our accuracies are higher than existing predictors which use only sequence information. Since such information as gene ontology can be obtained only for known proteins, our predictor is considered to be useful for subcellular location prediction of newly-discovered proteins. Furthermore, the idea of combination of alignment and amino acid frequency is novel and general so that it may be applied to other problems in bioinformatics. Our method for plant is also implemented as a web-system and available on .

## Background

**Figure 1:**
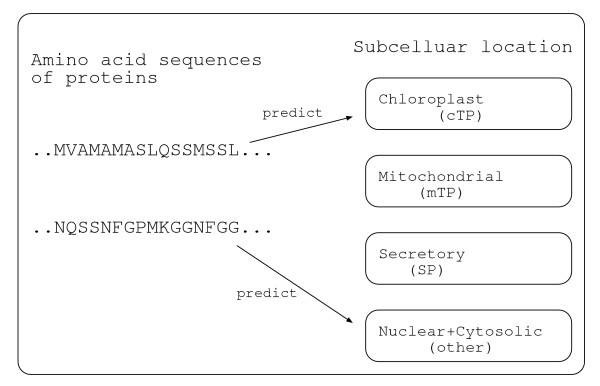
**Subcellular location prediction of proteins**. Subcellular location prediction of proteins is a problem of predicting which part in a cell a given protein is transported to, where an amino acid sequence of the protein is given as an input.

Predicting subcellular location of proteins is one of the major problems in bioinformatics. This is a problem of predicting which part (e.g., Mitochondria, Chloroplast, etc.) in a cell a given protein is transported to, where an amino acid sequence (i.e., string data) of the protein is given as an input as shown in Fig. 1. This problem is becoming more important since information on subcellular location is helpful for annotation of proteins and genes and the number of complete genomes is rapidly increasing. Many methods have been proposed using various computational techniques. Furthermore, many web-based prediction systems have been developed based on these proposed methods.

PSORT [1,2] is historically the first subcellular location predictor. PSORT and its major extension, such as WoLF PSORT [3,4], use various sequence-derived features such as the presence of sequence motifs and amino acid compositions. Although there are many predicting methods, they can be roughly classified into two groups. One is the N-terminal based method and the other is based on amino acid composition. TargetP [5] requires the N-terminal sequence as an input into two layers of artificial neural networks (ANN), utilizing the earlier binary predictors, SignalP [6] and ChloroP [7]. Reczko and Hatzigeorgiou used a bidirectional recurrent neural network with the first 90 residues in the N-terminal sequence [8].

Cedano et al. developed ProtLock [9], which is based on the amino acid composition and the least Mahalanobis distance algorithm. Chou and Elrod used the covariant discriminant algorithm besides amino acid composition [10]. NNPSL [11] is an ANN-based method using the amino acid composition by Reinhardt and Hubbard. After the successful report by Reinhardt and Hubbard [11], application of machine learning techniques became popular in this field. A support vector machine (SVM) was implemented for SubLoc [12] instead of the ANN. Incorporating amino acid order as well as amino acid composition is expected to make it possible to improve prediction performance. The pseudo-amino acid composition was proposed by Chou [13] in order to deal with the effect of the amino acid order. Moreover, Cai and Chou [14] have recently developed an accurate method integrating the pseudo-amino acid composition, the gene ontology information [15], and the functional domain composition [16]. Park and Kanehisa [17] developed an efficient method that incorporates compositions of dipeptides and gapped amino acid pairs besides the conventional amino acid composition. Yu *et al.*[18] successfully predicted localizations for Gram-negative bacteria by multiple feature vectors [19] based on *n*-peptide compositions. The concepts of the pseudo-amino acid and gapped amino acid pair compositions were merged in the residue-couple model proposed by [20]. Nair and Rost introduced LOCtree and showed mimicking the mechanism of cellular sorting yields good accuracies [21].

Recently, Matsuda *et al.*[22] proposed a novel representation of protein sequences. That representation involves local amino acid compositions and twin amino acids, and local frequencies of distance between successive (basic, hydrophobic, and other) amino acids. Each sequence is split into three parts: N-terminal, middle, and C-terminal in order to calculate the local features. The N-terminal part is further divided into four regions for considering ambiguity in the length and position of signal sequences. It was combined with SVM for prediction of subcellular location of proteins. The results of computational experiments suggest that their method is one of the state-of-the-art methods. Though the prediction accuracy is high, the method is based on various heuristics. Furthermore, many of the heuristics are specific to the protein subcellular location problem. In this paper, we try to develop a less heuristic method for the protein subcellular location problem and improve the prediction accuracies. Our method does not use any knowledge of motifs, gene ontologies and other databases. Furthermore, feature vector is very simple, that is amino acid composition of 20 elements. Development of such a method is important since it may be applied to other problems in bioinformatics. For example, the *spectrum kernel *[23], which is a simple and general kernel function for SVM, has been applied to various problems including remote homology detection [23], recognition of DNA-binding proteins [24], prediction of protein-protein interactions [25] and prediction of protein subcellular location.

To develop a general and more accurate method, we combine two-techniques in a simple but novel and general way: sequence alignment and a feature vector based on amino acid composition. Although there are predictors (e.g., PSLpred [26], ESLpred [27]) which combine sequence similarity and SVM based method, alignment is applied directly to amino acid sequences. On the other hand, in our method as explained later, alignment is applied to block sequences, in which each block has an amino acid composition-based feature vector. Furthermore, although there are some predictors which utilize sequence similarity and amino acid compositions (e.g. [28]), their method is a kind of hybrid procedure. That is, if sequence alignment can identify some homologous sequence, the prediction by sequence alignment is used. Otherwise, the prediction by SVM based method is used. Therefore, in their method, sequence alignment and SVM based method are independently executed. However, in our method, results of alignment are used as elements of kernel matrix.

**Figure 2:**
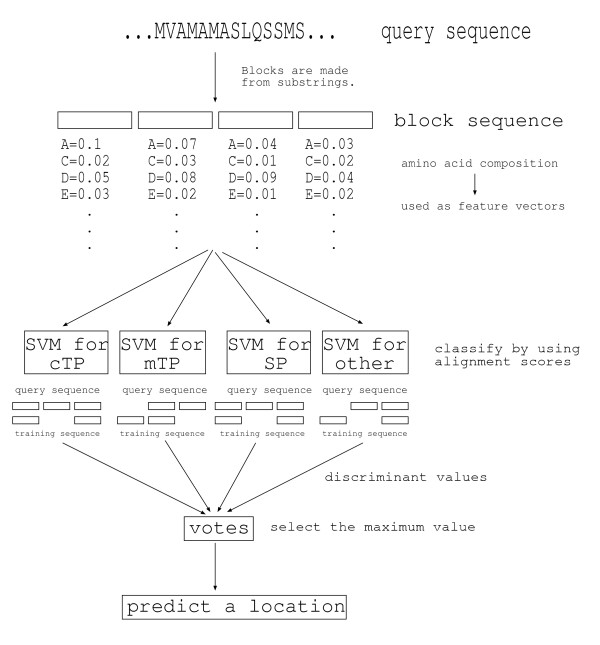
**Overview of our method**. Elements of our proposed kernel matrix are scores of alignment between block sequences of proteins. The alignment scores are calculated in which blocks are treated as if these were residues and the score between blocks is calculated from the corresponding feature vectors based on the amino acid frequency.

It should be noted that amino acid composition-based feature vector is almost the same as spectrum kernel. Elements of our proposed kernel matrix are scores of alignment between sequences of substrings of proteins. The alignment scores are calculated in accordance with amino acid composition-based feature vectors as shown in Fig. 2 (Details are explained later). As a result, we succeeded in improving prediction accuracies of the protein subcellular location problem for plant. To evaluate the prediction accuracies, we extracted plant data sets from TargetP data base and compared our method with existing methods through fivefold cross validation tests. Although our prediction method is less specialized for localization prediction than existing predictors, the overall accuracy and average MCC, which are standard measures of prediction accuracy, are 0.9096 and 0.8655 respectively. They are higher than existing predictors which use only sequence information. Furthermore, our method for plant is implemented as a web-system and available on [29].

Since the datasets obtained from TargetP is not very new, we also applied our method to plant datasets included in WoLF PSORT package version 0.2 that can be downloaded from [30]. As a result the overall accuracy of 0.8703 was obtained and this value is higher than that of WoLF PSORT.

## Results

Table 1 shows the comparison of predictive accuracies on the TargetP plant data sets with existing methods which use only sequence information. It is seen that MCC of our proposed method for "cTP" is higher than those of the other three methods by 0.081 ~ 0.131. It is also seen that MCC for "mTP" is higher than those of the other three methods by 0.051 ~ 0.104. Since 0.05 is considered to be a large improvement, our approach seems to be suitable for predicting locations of plant proteins which destine for "cTP" and "mTP". On the other hand, improvements of MCC for "SP" and "other" are 0.028 ~ 0.041 and 0.003 ~ 0.080 respectively. Although these improvements are not large, our sensitivity and specificity for "SP" are very high (0.9665 and 0.9319 respectively). Thus, our method is also suitable for predicting the location of proteins which destine for "SP" although the improvements are small. Therefore, our method succeeded in improving accuracies for "cTP" and "mTP" substantially and exceeding good accuracies of existing methods for "SP". However, our method is less effective at improving accuracies for "other" although our MCC for "other" is still higher than existing predictors in Table 1.

**Table 1:** Comparison of predictive accuracy for plant proteins in the TargetP data set. "cTP", "mTP", "SP", and "other" indicate proteins destined for chloroplast, mitochondria, secretory pathway, and other locations (nucleus and cytosol), respectively.

Predictor	Location	Sensitivity	Specificity	MCC	Average MCC	Overall Accuracy
Our method	cTP	0.8440	0.9015	0.8507	0.8655	0.9096
	mTP	0.9348	0.9125	0.8735		
	SP	0.9665	0.9319	0.9282		
	other	0.8148	0.8684	0.8095		

Matsuda et al. (2005)	cTP	0.7591	0.8474	0.7694	0.8244	0.8809
	mTP	0.9240	0.8652	0.8227		
	SP	0.9219	0.9326	0.8983		
	other	0.8210	0.8586	0.8070		

Kim et al. (2004)	cTP	0.6874	0.8435	0.7222	0.7791	0.8479
	mTP	0.8970	0.8392	0.7773		
	SP	0.8592	0.9428	0.8872		
	other	0.8027	0.7549	0.7296		

Emanuelsson et al. (2000)	cTP	0.85	0.69	0.72	0.79	0.853
	mTP	0.82	0.90	0.77		
	SP	0.91	0.95	0.90		
	other	0.85	0.78	0.77		

**Note: **Parameters of Table 2 are used in our method.

It is to be noted that our overall accuracy and average MCC are higher than any other predictor in Table 1. Furthermore, our MCC for each location is higher than that of any other predictor. Thus, it can be said that the prediction accuracy of our method is higher than existing predictors which use only sequence information.

Table 2 shows the values of parameters used in this experiment. Gap penalty denotes the penalty per gap in alignment between block sequences. That is, the score of -0.4 is given to a pair of a block and a gap. *γ *is the parameter of RBF kernel. "posconstraint" is a parameter of GIST (see "Methods") which sets an explicit upper bound on the magnitude of the weights for positive training examples. Similarly, "negconstraint" sets an explicit upper bound on the magnitude of the weights for negative training examples. "c" and "w" are used to transform given protein sequences into block sequences as explained later.

**Table 2:** Parameters used in the computational experiment. "Posconstraint" and "negconstraint" are parameters used in GIST.

Location	Gap penalty	*γ *of RBF	Posconstraint	Negconstraint	c	w
cTP	0.4	2.5	1.000000	0.009853	10	20
mTP	0.4	2.5	0.151072	0.099261	10	20
SP	0.4	2.5	0.032387	0.008566	10	20
Other	0.4	2.5	0.039955	0.008566	10	20

Since data sets of TargetP are not very new, we also applied our method to plant data sets of WoLF PSORT, which are included in WoLF PSORT package version 0.2 and can be downloaded from [30]. We removed 35 proteins which have dual locations from original 2426 proteins. As a result, obtained average MCC and overall accuracy were 0.8343 and 0.8703 respectively. Although we have not implemented for data sets which include dual location proteins, our overall accuracy would still be 0.8557 even if our method predicted a false location for every dual location protein. Since the overall accuracy of WoLF PSORT is 0.86, it seems that our method is also efficient for relatively new data sets. Details are shown in Table 3. Furthermore, we also applied our method to TargetP non-plant data sets. Details are shown in Table 4. Obtained average MCC and overall accuracy are 0.8452 and 0.9204 respectively. Although these values are slightly worse than those of Matsuda et al. [22], these are better than the other predictors of Table 4. Our MCC for mTP and "other" are worse than [22] by 0.022 and 0.005 respectively although our MCC for SP is slightly better than [22].

**Table 3:** Prediction accuracy for WoLF PSORT plant data sets. "Chlo," "cyto," "cysk," "E.R.," "extr," "golg," "mito," "nucl," "pero," "plas," "vacu" indicate, chloroplast, cytosol, cytoskeleton, endoplasmic reticulum, extracellular, Golgi apparatus, mitochondria, nuclear, peroxisome, plasma membrane, vacuolar membrane respectively.

Predictor	Location	No. of seq.s	Sensitivity	Specificity	MCC	Average MCC	Overall Accuracy
Our method (2391 sequences)	chlo	750	0.9733	0.8795	0.8893	0.8343	0.8703
	cyto	432	0.9329	0.8258	0.8491		
	cysk	41	0.7561	1.0000	0.8677		
	E.R.	69	0.7391	0.9623	0.8395		
	extr	114	0.8246	0.7581	0.7797		
	golg	29	0.6207	0.9474	0.7645		
	mito	210	0.7857	0.9066	0.8303		
	nucl	456	0.8509	0.8788	0.8329		
	pero	52	0.7500	0.9512	0.8417		
	plas	165	0.7212	0.9675	0.8255		
	vacu	73	0.7671	0.9655	0.8569		

Horton et al. (2006) (2426 sequences)							0.86

**Note: **In our method, although gap penalty = 0.4, *γ *= 2.5, *c *= 10, *w *= 20 are given, posconstraint and negconstraint are not specified.

**Table 4:** Comparison of predictive accuracy for non-plant proteins in the TargetP data set. "mTP", "SP", and "other" indicate proteins destined for mitochondria, secretory pathway, and other locations (nucleus and cytosol), respectively.

Predictor	Location	Sensitivity	Specificity	MCC	Average MCC	Overall Accuracy
Our method	mTP	0.7647	0.8990	0.8005	0.8452	0.9204
	SP	0.9157	0.9056	0.8790		
	other	0.9576	0.9324	0.8560		

Matsuda et al. (2005)	mTP	0.8303	0.8635	0.8228	0.8542	0.9229
	SP	0.9091	0.9118	0.8788		
	other	0.9498	0.9409	0.8609		

Kim et al. (2004)	mTP	0.6483	0.8569	0.7121	0.7635	0.8762
	SP	0.8530	0.8736	0.8158		
	other	0.9389	0.8819	0.7626		

Emanuelsson et al. (2000)	mTP	0.89	0.67	0.73	0.8233	0.900
	SP	0.96	0.92	0.92		
	other	0.88	0.97	0.82		

Reczko et al. (2004)	mTP	0.78	0.82	0.77	0.8333	0.913
	SP	0.93	0.91	0.89		
	other	0.93	0.94	0.84		

**Note: **In our method, although gap penalty = 0.4, *γ *= 2.5, *c *= 10, *w *= 20 are given, posconstraint and negconstraint are not specified.

## Control experiments

We executed some control experiments in order to evaluate the results of our method. Table 5 shows the comparison of our methods which use following feature vectors: (i) amino acid composition (ii) all adjacent amino acid composition (iii) amino acid composition and twin amino acid composition. "twin amino acid composition" is defined as the frequency of length two substrings "XX" for any amino acid X. For example, if an amino acid sequence "AAACC" is given, AA = 0.5, CC = 0.25 and the others = 0 are obtained. As a result, (i) yielded the best result, 90.96% of overall accuracy. Overall accuracy of (ii) and (iii) were 73.72% and 90.64% respectively. Although twin amino acids were effective for the method of Matsuda *et al.*[22], they did not improve the overall accuracy of our method.

**Table 5:** Comparison of predictive accuracy for plant proteins in the TargetP data set with three types of feature vectors

Predictor	Location	Sensitivity	Specificity	MCC	Average MCC	Overall Accuracy
20 feature vectors (amino acid composition)	cTP	0.8440	0.9015	0.8507	0.8655	0.9096
	mTP	0.9348	0.9125	0.8735		
	SP	0.9665	0.9319	0.9282		
	other	0.8148	0.8684	0.8095		

400 feature vectors (all adjacent amino acid composition)	cTP	0.8227	0.4128	0.4806	0.6126	0.7372
	mTP	0.8342	0.8797	0.7686		
	SP	0.8253	0.8880	0.8015		
	other	0.2963	0.8000	0.4339		

40 feature vectors	cTP	0.8014	0.9040	0.8270	0.8594	0.9064
	mTP	0.9402	0.9081	0.8739		
	SP	0.9628	0.9317	0.9255		
	other	0.8272	0.8590	0.8110		

**Note: **Parameters of Table 2 are used for all methods.

We also implemented the nearest neighbor classifier (NNC) with the similarity measure proposed in this manuscript. The obtained average MCC and overall accuracy were 0.7255 and 0.8128. These values are worse than the SVM based method by 0.14 and 0.0968 respectively. Therefore, in the control experiment where the same similarity measure (our proposed method) and different classifiers (SVM and NNC) are used, SVM was better than NNC. Details are shown in the first and third methods of Table 6.

**Table 6:** Comparison of predictive accuracy of our methods for plant proteins in the TargetP data set. "cTP", "mTP", "SP", and "other" indicate proteins destined for chloroplast, mitochondria, secretory pathway, and other locations (nucleus and cytosol), respectively.

Predictor	Location	Sensitivity	Specificity	MCC	Average MCC	Overall Accuracy
Our method (SVM with specifying posconstraint and negconstraint)	cTP	0.8440	0.9015	0.8507	0.8655	0.9096
	mTP	0.9348	0.9125	0.8735		
	SP	0.9665	0.9319	0.9282		
	other	0.8148	0.8684	0.8095		

Our method (SVM without specifying posconstraint and negconstraint)	cTP	0.7518	0.9550	0.8249	0.8525	0.8989
	mTP	0.9592	0.8506	0.8363		
	SP	0.9554	0.9554	0.9375		
	other	0.7963	0.8897	0.8111		

Nearest Neighbor Classifier (with our similarity measure)	cTP	0.7447	0.7664	0.7131	0.7255	0.8128
	mTP	0.8750	0.7854	0.7098		
	SP	0.8848	0.9015	0.8508		
	other	0.6111	0.7674	0.6284		

**Note: **In the first method, all parameters of Table 2 are given. In the second and third methods, gap penalty = 0.4, *γ *= 2.5, *c *= 10, *w *= 20 are given although posconstraint and negconstraint are not specified.

Furthermore, according to Matsuda et al. (2005), the overall accuracy of NNC with Smith-Waterman score is 75.7 %. Therefore, in the control experiment where the different similarity measures (our proposed method and Smith-Waterman score) and the same classifier (NNC) are used, our proposed similarity measure was better than Smith-Waterman score by 0.0558.

Mitopred [31] is a web server which enables prediction of nucleus encoded mitochondrial proteins in all eukaryotic species. We input plant proteins of TargetP into Mitopred and compared the accuracies with our method. Table 7 shows the comparison of our method and Mitopred of each confidence cutoff. When confidence cutoff is 60%, Mitopred yielded its best MCC of 0.7816. However, our MCC is 0.8587 and higher than that of Mitopred by 0.0771.

**Table 7:** Comparison of predictive accuracy of our method and Mitopred for plant proteins in the TargetP data set

Predictor	Location	Sensitivity	Specificity	MCC
Our method	mTP	0.9348	0.8958	0.8587
Mitopred (60%)	mTP	0.9348	0.8132	0.7816
Mitopred (85%)	mTP	0.5815	0.8807	0.5918
Mitopred (99%)	mTP	0.0598	0.8148	0.1492

**Note: **Parameters of Table 2 are used for our method.

Furthermore, MitPred is a web-server specifically trained to predict the proteins which are destined to localized in mitochondria in yeast and animals [32]. We applied MitPred to both plant and nonplant proteins of TargetP. MitPred has three types of methods; SVM method, BLAST+SVM method and HMM+SVM method. For both plant and nonplant datasets, BLAST+SVM method yielded the better MCC than any other method of MitPred. For plant dataset, MCC of BLAST+SVM method of MitPred was 0.8158, which is less than MCC of our method by 0.0429. On the other hand, for nonplant dataset, MCC of BLAST+SVM method of MitPred was 0.9268, which is better than MCC of our method by 0.1642. Since MitPred is specifically trained to predict the mitochondrial proteins in yeast and animals, the accuracy for nonplant mTP is better than ours. However, our accuracy is better than that of MitPred for plant although our prediction method is not specialized to mTP. Table 8 shows the detailed comparison of our method and MitPred.

**Table 8:** Comparison of predictive accuracy of our method and MitPred for both plant and nonplant proteins in the TargetP data set

Predictor	Location	Sensitivity	Specificity	MCC
Our method	plant mTP	0.9348	0.8958	0.8587
MitPred (SVM)	plant mTP	0.8234	0.8584	0.7418
MitPred (BLAST+SVM)	plant mTP	0.9429	0.8422	0.8158
MitPred (HMM+SVM)	plant mTP	0.8668	0.8484	0.7644

Our method	nonplant mTP	0.7547	0.8333	0.7626
MitPred (SVM)	nonplant mTP	0.8194	0.8863	0.8302
MitPred (BLAST+SVM)	nonplant mTP	0.9380	0.9355	0.9268
MitPred (HMM+SVM)	nonplant mTP	0.8571	0.9408	0.8830

**Note: **In our methods, parameters shown in Table 2 of the manuscript were used, but "posconstraint" and "negconstraint" were not specified for nonplant mTP. For MitPred, we used default parameters as follows. In SVM method, threshold was set as 0.5. E-value cutoff of BLAST+SVM method was 1e-4. E-value and SVM threshold for HMM based Pfam search+SVM method were 1e-5 and 0.5 respectively.

By utilizing BLASTclust, we obtained non-redundant dataset at 70% and 25% from TargetP plant data set. Table 9 shows numbers of sequences for each location. By applying our SVM based method to these data sets, overall accuracy of 0.8625 and 0.7829 were obtained respectively. Therefore, it can be said that our method is efficient even when sequence similarity is relatively small. Details are shown in Table 10.

**Table 9:** Numbers of sequences of non-redundant datasets at 25% and 70 % obtained from TargetP plant data sets by utilizing BLASTclust

Subcellular location	No. of sequences (plant) (non-redundant at 25%)	No. of sequences (plant) (non-redundant at 70%)
Chloroplast(cTP)	95	116
Mitochondrial(mTP)	200	314
Secretory(SP)	119	232
Nuclear+cytosolic(other)	125	138

Total	539	800

**Table 10:** Predictive accuracies of our method for datasets of Table 9

Predictor	Location	Sensitivity	Specificity	MCC	Average MCC	Overall Accuracy
Our method (25%)	cTP	0.7684	0.6577	0.6434	0.7068	0.7829
	mTP	0.7900	0.8404	0.7111		
	SP	0.7311	0.9158	0.7751		
	other	0.8320	0.7172	0.6976		

Our method (70%)	cTP	0.6638	0.8750	0.7289	0.7937	0.8625
	mTP	0.9618	0.8118	0.8006		
	SP	0.9095	0.9505	0.9020		
	other	0.7246	0.8475	0.7431		

**Note: **Parameters of Table 2 are used for all methods.

## Discussion

We proposed a novel subcellular location predicting method which is based on sequence alignment and amino acid composition. Through fivefold cross validation tests for TargetP plant data sets, we obtained the overall accuracy of 0.9096 and the average MCC of 0.8655. These values are higher than existing predictors which use only sequence information. We believe that the high accuracy attained by our method indicates that our alignment algorithm is automatically detecting signal sequences. Localization signals, such as the mitochondrial transit signal are highly diverse at the amino acid level, but share some features such as regions of positive charge or amphiphilic nature. Thus by aligning the amino acid compositions of small blocks, instead of individual amino acids, our technique may capture some features of localization signals such as transit signals.

**Figure 3:**
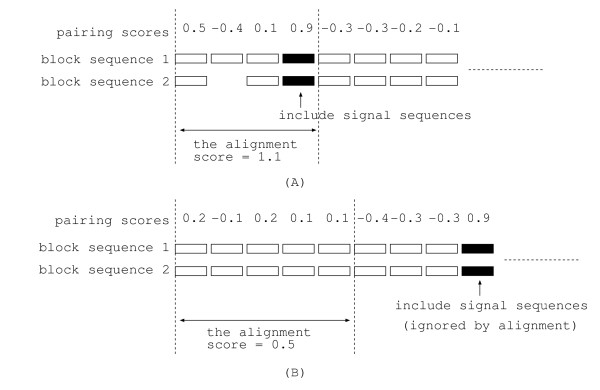
**Global and local alignment**. Global alignment is applied to left ends of block sequences and local alignment is applied to right ends of block sequences. (A) The alignment detects the signal sequence which is in the left part of sequences. (B) The alignment does not detect the signal sequence since the alignment score decreases if the block of the signal sequence is included.

As mentioned in the previous section, our method is effective for "cTP", "mTP" and "SP," but less effective for "other". The reason is discussed in the following. In general, proteins destined for chloroplast, mitochondria, and secretory pathway have signal sequences in their *N *termini. On the other hand, proteins destined for nucleus and cytosol have one or more signal sequences in the middle part of their sequence. As explained later, global alignment is applied to left ends of sequences and local alignment is applied to right ends of sequences in our method as shown in Fig. 3. Then, it detects signal sequences in *N *termini with higher probability than in the middle part of sequences. This is the reason why our method cannot improve the accuracy for "other" substantially. Furthermore, conventional local alignment, in which local alignment is applied to both ends of sequences, did not improve the prediction accuracy in our preliminary experiment. This fact is reasonable since conventional local alignment ignores signal sequences in *N*-termini with higher probability than our method.

Although the above discussion can also be applied to SP and the "other" subset of non-plant datasets of TargetP, the result for non-plant mTP seems to be contradicting to this. We think the reason is either due to the influence of not using distance frequency [22], or that the composition of non-plant mTP sequences is only 13.6%. Alternatively, there may exist some unknown signal sequences relating to subcellular location in N-termini of non-plant mTP proteins. However, it should be noted that the difference of accuracies between our method and [22] is very small (0.25%).

Parameters in Table 2 were determined by trial and error approach, because it is very important to assign appropriate values to them. Since our feature vector consists of only amino acid composition, the information of subcellular location signals may be disappeared if *w *is too large or too small. Even when appropriate *w *is given, the information of signals may be divided and ignored if the edge of the window is in the middle of a signal sequence. However, such ignored signals can be found if *c *is set appropriately. *c *= *w*/2 is considered to be one of the best relationships between *c *and *w*.

Assigning appropriate values to *γ *and gap penalty is also important. As explained later, the pairing score in alignment of our method takes a positive value when two blocks are similar to each other. On the other hand, the pairing score takes a negative value when two blocks are not similar. However, if *γ *is too large, the pairing score takes a negative value in most cases. On the other hand, if *γ *is too small, the pairing score takes a positive value in most cases. Therefore, it is very important to set appropriate *γ *since it determines the threshold of "whether blocks are similar to each other". The value of gap penalty also strongly influences the result of alignment. The detailed method of determining these parameters are described in the following.

Let *p *represent the gap penalty. We examined *p *= 0, 0.2, 0.4, 0.6, 0.8 1.0, *γ *= 0.3, 0.6, 1.0, 1.5, 2.0, 2.5, 3.0, 3.5 and (*w*, *c*) = (40, 20), (30, 15), (20, 10) respectively. Among values above, (*p*, *γ*, *w*, *c*) = (0.4, 2.5, 20, 10) yielded the best accuracy. Since we did not examine so many values, the accuracy may be improved by further optimization for these values. We believe that further optimization for (*w*, *c*) is more hopeful than those for *p *and *γ *to improve the accuracy. It is to be noted that the same values were used for (*p*, *γ*, *w*, *c*) in all the experiments in this paper.

In terms of "posconstraint" and "negconstraint", the first and second methods of Table 6 show the comparison of

• (1) our method with specifying "posconstraint" and "negconstraint,"

• (2) our method without specifying "posconstraint" and "negconstraint."

Although the average MCC and overall accuracy of (1) are better than those of (2) by 0.013 and 0.0107 respectively, (2) is better than the other predictors shown in Table 1. In terms of SP, MCC of (2) is better than (1) by 0.0093 although (1) is better than (2) for the other locations. Thus, our predictor can yield good accuracies even when "posconstraint" and "negconstraint" are not specified.

To optimize these parameters used in (1), we set *posconstraint *= *exp*(-0.07·*i*) and *negconstraint *= *exp*(-0.07·*j*) and scanned *i *and *j *from 0 to 99. Then, values which yielded the best overall accuracy were used for (1) and are shown in Table 2. We believe that further optimization for "posconstraint" and "negconstraint" does not substantially improve the accuracies.

**Figure 4:**
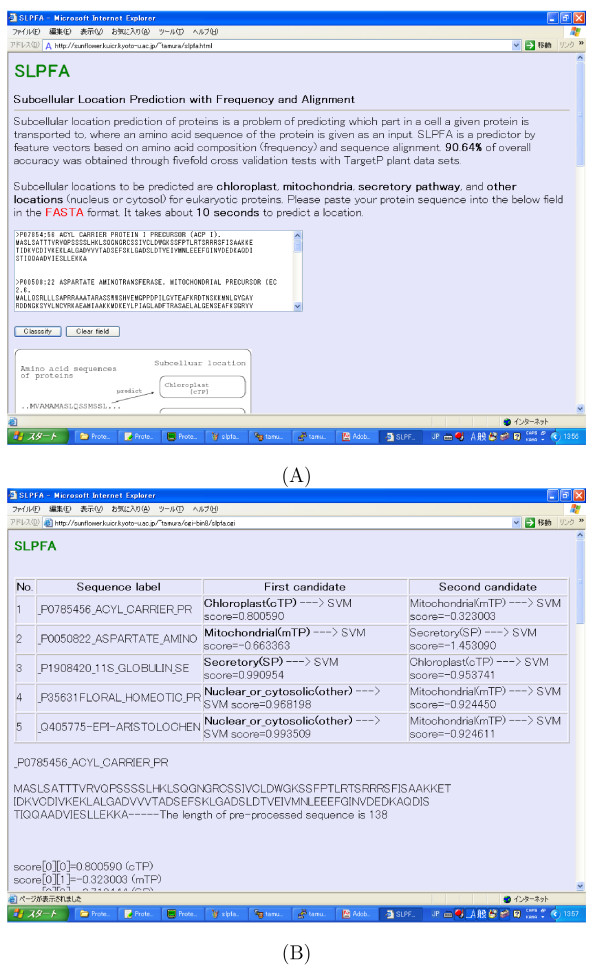
**Web-based system**. Our proposed method is implemented as a web-system and available on [29]. (A) Amino acid sequences in the FASTA format are pasted into our web-system as input. (B) Two candidate locations are shown for each input sequence along with scores.

We also developed a web-based prediction system based on our proposed method for plant. It is available on [29]. When amino acid sequences in the FASTA format are given as shown in Fig. 4(A), the web-system returns the first and second candidates of the location and their scores as shown in Fig. 4(B). Although an overall accuracy of 90.96% was achieved in our five-fold cross validation tests, it takes about 10 seconds to predict a location. The reason why our web-system is not fast is that it calculates similarity scores between every input sequence and all training sequences. However, these calculations can be parallelized. If there are enough CPUs, the calculation time would be in a second although our web-system is not parallelized so far.

## Conclusion

Our overall accuracy, average MCC and MCC for each location are higher than any other predictor which use sequence information for plant proteins of TargetP. Furthermore, our overall accuracy for plant proteins of WoLF PSORT is higher than WoLF PSORT although used datasets are slightly different. Therefore, it can be said that our prediction method for plant is efficient and succeeded in improving accuracies of existing methods. Although it is known that the overall accuracies (0.923) of the predictor for plant by Chou and Cai [15,16] are slightly higher than those of our predictor, their method uses the information of gene ontology and functional domain. Since such information can be obtained only for known proteins, it is still important to develop a predictor which use only sequence information of amino acids. Thus, our predictor is considered to be useful for subcellular location prediction of newly-discovered proteins. Furthermore, the idea of combination of alignment and amino acid frequency is novel and general so that it may be applied to other problems in bioinformatics. Although our web-based predictor is now available for only plant, constructing the system for non-plant and plant which includes more numbers of subcellular locations is our on-going work.

## Methods

Our proposed prediction method uses support vector machines (SVMs). In order to apply SVMs to classification of sequence data, it is usually required to define a kernel function between sequences. In our proposed method, the kernel value is the alignment score between two sequences. However, the alignment score is different from usual ones (e.g., Smith-Waterman score). We did not use the score matrix such as PAM or BLOSUM. In our proposed method, each input sequence is transformed into a sequence of blocks, where a block is a fixed-length consecutive substring of the input sequence. Then, in the alignment procedure, each block is treated as if it were a residue. The score between two blocks is calculated from features vectors of these blocks, where a feature vector of a block is based on the amino acid composition of the block.

**Figure 5:**
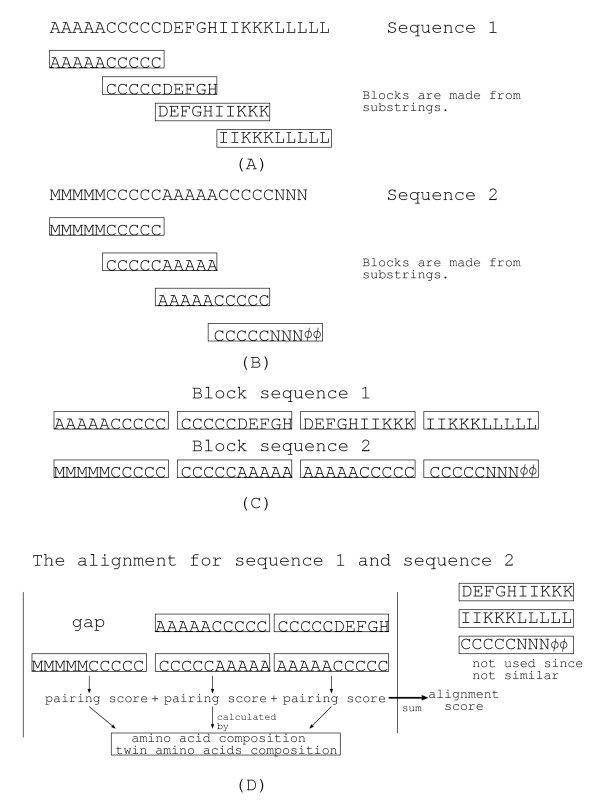
**Alignment for block sequences**. (A)(B)(C) Transformation of the input sequences into block sequences, where *w *= 10 is the length of blocks and *c *= 5 is the distance between neighboring blocks. (D) Alignment of block sequences. While global alignment is applied to left ends of block sequences, local alignment is applied to right ends of block sequences.

Assume that *sequence*1 = *AAAAACCCCCDEFGHIIKKKLLLLL *and *sequence*2 = *MMMMMCCCCCAAAAACCCCCNNN *are given as shown in Figures 5(A) and 5(B) respectively. We transform *sequence*1 (resp. *sequence*2) into a sequence of four blocks as in Fig. 5(A) (resp. Fig. 5(B)). Each block is of a fixed length *w *and two consecutive blocks can overlap. The distance (in the number of residues) between two consecutive blocks is denoted by *c*. Note that *w *= 10 and *c *= 5 are used in Figures 5(A) and 5(B). In Fig. 5(B), "*φ*"s are assigned to the rightmost block since there are no corresponding amino acids. A feature vector is calculated for each block. Our feature vector consists of a composition of 20 amino acids. For example, the amino acid compositions for *AAAAACCCCC*, *DEFGHIIKKK*, and *CCCCCNNNφφ *are (A = 5/10, C = 5/10, the other = 0), (D = 1/10, E = 1/10, F = 1/10, G = 1/10, H = 1/10, I = 2/10, K = 3/10, the other = 0), and (C = 5/10, N = 3/10, the other = 0) respectively in our method.

To calculate the alignment score, we have to define the score between blocks. For example, the score of alignment in Fig. 5(D) is calculated as the sum of the following.

• a pairing score between a gap and MMMMMCCCCC (a gap penalty)

• a pairing score between AAAAACCCCC and CCCCCAAAAA

• a pairing score between CCCCCDEFGH and AAAAACCCCC

We use 2·*exp*(-*γ*||bx,jx
 MathType@MTEF@5@5@+=feaafiart1ev1aaatCvAUfKttLearuWrP9MDH5MBPbIqV92AaeXatLxBI9gBaebbnrfifHhDYfgasaacPC6xNi=xH8viVGI8Gi=hEeeu0xXdbba9frFj0xb9qqpG0dXdb9aspeI8k8fiI+fsY=rqGqVepae9pg0db9vqaiVgFr0xfr=xfr=xc9adbaqaaeGacaGaaiaabeqaaeqabiWaaaGcbaacbeGae8Nyai2aaSbaaSqaaiabdIha4jabcYcaSiabdQgaQnaaBaaameaacqWG4baEaeqaaaWcbeaaaaa@32B9@ - by,jy
 MathType@MTEF@5@5@+=feaafiart1ev1aaatCvAUfKttLearuWrP9MDH5MBPbIqV92AaeXatLxBI9gBaebbnrfifHhDYfgasaacPC6xNi=xH8viVGI8Gi=hEeeu0xXdbba9frFj0xb9qqpG0dXdb9aspeI8k8fiI+fsY=rqGqVepae9pg0db9vqaiVgFr0xfr=xfr=xc9adbaqaaeGacaGaaiaabeqaaeqabiWaaaGcbaacbeGae8Nyai2aaSbaaSqaaiabdMha5jabcYcaSiabdQgaQnaaBaaameaacqWG5bqEaeqaaaWcbeaaaaa@32BD@||^2^) - 1 as a pairing score between two blocks where bx,jx
 MathType@MTEF@5@5@+=feaafiart1ev1aaatCvAUfKttLearuWrP9MDH5MBPbIqV92AaeXatLxBI9gBaebbnrfifHhDYfgasaacPC6xNi=xH8viVGI8Gi=hEeeu0xXdbba9frFj0xb9qqpG0dXdb9aspeI8k8fiI+fsY=rqGqVepae9pg0db9vqaiVgFr0xfr=xfr=xc9adbaqaaeGacaGaaiaabeqaaeqabiWaaaGcbaacbeGae8Nyai2aaSbaaSqaaiabdIha4jabcYcaSiabdQgaQnaaBaaameaacqWG4baEaeqaaaWcbeaaaaa@32B9@ and bx,jx
 MathType@MTEF@5@5@+=feaafiart1ev1aaatCvAUfKttLearuWrP9MDH5MBPbIqV92AaeXatLxBI9gBaebbnrfifHhDYfgasaacPC6xNi=xH8viVGI8Gi=hEeeu0xXdbba9frFj0xb9qqpG0dXdb9aspeI8k8fiI+fsY=rqGqVepae9pg0db9vqaiVgFr0xfr=xfr=xc9adbaqaaeGacaGaaiaabeqaaeqabiWaaaGcbaacbeGae8Nyai2aaSbaaSqaaiabdIha4jabcYcaSiabdQgaQnaaBaaameaacqWG4baEaeqaaaWcbeaaaaa@32B9@ are feature vectors for blocks. Since 2·*exp*(-*γ*||bx,jx
 MathType@MTEF@5@5@+=feaafiart1ev1aaatCvAUfKttLearuWrP9MDH5MBPbIqV92AaeXatLxBI9gBaebbnrfifHhDYfgasaacPC6xNi=xH8viVGI8Gi=hEeeu0xXdbba9frFj0xb9qqpG0dXdb9aspeI8k8fiI+fsY=rqGqVepae9pg0db9vqaiVgFr0xfr=xfr=xc9adbaqaaeGacaGaaiaabeqaaeqabiWaaaGcbaacbeGae8Nyai2aaSbaaSqaaiabdIha4jabcYcaSiabdQgaQnaaBaaameaacqWG4baEaeqaaaWcbeaaaaa@32B9@ - by,jy
 MathType@MTEF@5@5@+=feaafiart1ev1aaatCvAUfKttLearuWrP9MDH5MBPbIqV92AaeXatLxBI9gBaebbnrfifHhDYfgasaacPC6xNi=xH8viVGI8Gi=hEeeu0xXdbba9frFj0xb9qqpG0dXdb9aspeI8k8fiI+fsY=rqGqVepae9pg0db9vqaiVgFr0xfr=xfr=xc9adbaqaaeGacaGaaiaabeqaaeqabiWaaaGcbaacbeGae8Nyai2aaSbaaSqaaiabdMha5jabcYcaSiabdQgaQnaaBaaameaacqWG5bqEaeqaaaWcbeaaaaa@32BD@||^2^) - 1 always takes a value in [-1, 1], pairing scores also take a value in [-1, 1] in our implemented method. Note that the pairing score takes a positive value when two blocks are similar to each other. On the other hand, the pairing score takes a negative value when two blocks are not similar.

Finally, the highest alignment scores between every two block sequences are calculated by dynamic programming. In our method, while both left ends of block sequences must be used by the alignment, we do not have to use right ends of block sequences. In other words, global alignment is applied to left ends of block sequences and local alignment is applied to right ends of block sequences. For example, the best alignment between block sequences which are corresponding to *sequence*1 and *sequence*2 is shown in Fig. 5(D). Note that blocks *DEFGHIIKKK *and *IIKKKLLLLL *of *sequence*1, and a block *CCCCCNNNφφ *of *sequence*2 are not used in Fig. 5(D). Since 2·*exp*(-*γ*||bx,jx
 MathType@MTEF@5@5@+=feaafiart1ev1aaatCvAUfKttLearuWrP9MDH5MBPbIqV92AaeXatLxBI9gBaebbnrfifHhDYfgasaacPC6xNi=xH8viVGI8Gi=hEeeu0xXdbba9frFj0xb9qqpG0dXdb9aspeI8k8fiI+fsY=rqGqVepae9pg0db9vqaiVgFr0xfr=xfr=xc9adbaqaaeGacaGaaiaabeqaaeqabiWaaaGcbaacbeGae8Nyai2aaSbaaSqaaiabdIha4jabcYcaSiabdQgaQnaaBaaameaacqWG4baEaeqaaaWcbeaaaaa@32B9@ - by,jy
 MathType@MTEF@5@5@+=feaafiart1ev1aaatCvAUfKttLearuWrP9MDH5MBPbIqV92AaeXatLxBI9gBaebbnrfifHhDYfgasaacPC6xNi=xH8viVGI8Gi=hEeeu0xXdbba9frFj0xb9qqpG0dXdb9aspeI8k8fiI+fsY=rqGqVepae9pg0db9vqaiVgFr0xfr=xfr=xc9adbaqaaeGacaGaaiaabeqaaeqabiWaaaGcbaacbeGae8Nyai2aaSbaaSqaaiabdMha5jabcYcaSiabdQgaQnaaBaaameaacqWG5bqEaeqaaaWcbeaaaaa@32BD@||^2^) - 1 might take a negative value, there is a possibility that higher alignment scores are obtained in the case where right ends of sequences are not used than in the case where right ends of sequences must be used. For example, in Figures 5(C) and 5(D), pairing *DEFGHIIKKK *(or *IIKKKLLLLL*) and *CCCCCNNNφφ *takes a negative value in our implemented method since these are not similar to each other. The optimal scores of alignments are used as elements of the kernel matrix. Finally, our predictor selects a location whose "discriminant" value, which is calculated by "gist-classify" [33], is higher than any other location.

### Mathematical description of the method

Here we present a mathematical description of our proposed method in order to provide precise information. Let *c *and *w *be some positive integers used as parameters. Let Si=si,1si,2…si,niφφφ⋯
 MathType@MTEF@5@5@+=feaafiart1ev1aaatCvAUfKttLearuWrP9MDH5MBPbIqV92AaeXatLxBI9gBaebbnrfifHhDYfgasaacPC6xNi=xH8viVGI8Gi=hEeeu0xXdbba9frFj0xb9qqpG0dXdb9aspeI8k8fiI+fsY=rqGqVepae9pg0db9vqaiVgFr0xfr=xfr=xc9adbaqaaeGacaGaaiaabeqaaeqabiWaaaGcbaGaem4uam1aaSbaaSqaaiabdMgaPbqabaGccqGH9aqpcqWGZbWCdaWgaaWcbaGaemyAaKMaeiilaWIaeGymaedabeaakiabdohaZnaaBaaaleaacqWGPbqAcqGGSaalcqaIYaGmaeqaaOGaeSOjGSKaem4Cam3aaSbaaSqaaiabdMgaPjabcYcaSiabd6gaUnaaBaaameaacqWGPbqAaeqaaaWcbeaaiiGakiab=z8aMjab=z8aMjab=z8aMjabl+Uimbaa@484B@ be given protein sequences (*i *= 1, 2,⋯,*m*), where *m *is the number of sequences and *n*_*i *_is the length of *S*_*i*_. Let Bi=bi,1bi,2…bi,max⁡(⌈(ni−w)/c⌉,0)+1
 MathType@MTEF@5@5@+=feaafiart1ev1aaatCvAUfKttLearuWrP9MDH5MBPbIqV92AaeXatLxBI9gBaebbnrfifHhDYfgasaacPC6xNi=xH8viVGI8Gi=hEeeu0xXdbba9frFj0xb9qqpG0dXdb9aspeI8k8fiI+fsY=rqGqVepae9pg0db9vqaiVgFr0xfr=xfr=xc9adbaqaaeGacaGaaiaabeqaaeqabiWaaaGcbaGaemOqai0aaSbaaSqaaiabdMgaPbqabaGccqGH9aqpcqWGIbGydaWgaaWcbaGaemyAaKMaeiilaWIaeGymaedabeaakiabdkgaInaaBaaaleaacqWGPbqAcqGGSaalcqaIYaGmaeqaaOGaeSOjGSKaemOyai2aaSbaaSqaaiabdMgaPjabcYcaSiGbc2gaTjabcggaHjabcIha4naabmaabaWaaCWaaeaadaqadaqaaiabd6gaUnaaBaaameaacqWGPbqAaeqaaSGaeyOeI0Iaem4DaChacaGLOaGaayzkaaGaei4la8Iaem4yamgacaGLUJVaayz+4dGaeiilaWIaeGimaadacaGLOaGaayzkaaGaey4kaSIaeGymaedabeaaaaa@5529@ be a sequence of substrings of *S*_*i*_, where *b*_*i*,*j *_= *s*_*i*,*c*(*j*-1)+1_*s*_*i*,*c*(*j*-1)+2 _...*s*_*i*,*c*(*j*-1)+*w*_. Note that there is a possibility that *b*_*i*,*j *_includes *φ*.

Let *x *and *y *be integers which satisfy 1 ≤ *x*, *y *≤ *m*. Moreover, let *j*_*x *_and *j*_*y *_be integers which satisfy 1 ≤ *j*_*x *_≤ max([(*n*_*x *_- *w*)*/c*], 0) + 1 and 1 ≤ *j*_*y *_≤ max([(*n*_*y *_- *w*)/*c*], 0) + 1. Feature vectors (defined later) of bx,jx
 MathType@MTEF@5@5@+=feaafiart1ev1aaatCvAUfKttLearuWrP9MDH5MBPbIqV92AaeXatLxBI9gBaebbnrfifHhDYfgasaacPC6xNi=xH8viVGI8Gi=hEeeu0xXdbba9frFj0xb9qqpG0dXdb9aspeI8k8fiI+fsY=rqGqVepae9pg0db9vqaiVgFr0xfr=xfr=xc9adbaqaaeGacaGaaiaabeqaaeqabiWaaaGcbaGaemOyai2aaSbaaSqaaiabdIha4jabcYcaSiabdQgaQnaaBaaameaacqWG4baEaeqaaaWcbeaaaaa@32B3@ and by,jy
 MathType@MTEF@5@5@+=feaafiart1ev1aaatCvAUfKttLearuWrP9MDH5MBPbIqV92AaeXatLxBI9gBaebbnrfifHhDYfgasaacPC6xNi=xH8viVGI8Gi=hEeeu0xXdbba9frFj0xb9qqpG0dXdb9aspeI8k8fiI+fsY=rqGqVepae9pg0db9vqaiVgFr0xfr=xfr=xc9adbaqaaeGacaGaaiaabeqaaeqabiWaaaGcbaGaemOyai2aaSbaaSqaaiabdMha5jabcYcaSiabdQgaQnaaBaaameaacqWG5bqEaeqaaaWcbeaaaaa@32B7@ are denoted by bx,jx
 MathType@MTEF@5@5@+=feaafiart1ev1aaatCvAUfKttLearuWrP9MDH5MBPbIqV92AaeXatLxBI9gBaebbnrfifHhDYfgasaacPC6xNi=xH8viVGI8Gi=hEeeu0xXdbba9frFj0xb9qqpG0dXdb9aspeI8k8fiI+fsY=rqGqVepae9pg0db9vqaiVgFr0xfr=xfr=xc9adbaqaaeGacaGaaiaabeqaaeqabiWaaaGcbaacbeGae8Nyai2aaSbaaSqaaiabdIha4jabcYcaSiabdQgaQnaaBaaameaacqWG4baEaeqaaaWcbeaaaaa@32B9@ and by,jy
 MathType@MTEF@5@5@+=feaafiart1ev1aaatCvAUfKttLearuWrP9MDH5MBPbIqV92AaeXatLxBI9gBaebbnrfifHhDYfgasaacPC6xNi=xH8viVGI8Gi=hEeeu0xXdbba9frFj0xb9qqpG0dXdb9aspeI8k8fiI+fsY=rqGqVepae9pg0db9vqaiVgFr0xfr=xfr=xc9adbaqaaeGacaGaaiaabeqaaeqabiWaaaGcbaacbeGae8Nyai2aaSbaaSqaaiabdMha5jabcYcaSiabdQgaQnaaBaaameaacqWG5bqEaeqaaaWcbeaaaaa@32BD@ respectively. The kernel-like value between *B*_*x *_and *B*_*y*_, which is denoted by *K*(*B*_*x*_, *B*_*y*_), is calculated by the following dynamic programming (DP) procedure:

K(Bx,By)=max⁡jx,jyD(jx,jy),
 MathType@MTEF@5@5@+=feaafiart1ev1aaatCvAUfKttLearuWrP9MDH5MBPbIqV92AaeXatLxBI9gBaebbnrfifHhDYfgasaacPC6xNi=xI8qiVKYPFjYdHaVhbbf9v8qqaqFr0xc9vqFj0dXdbba91qpepeI8k8fiI+fsY=rqGqVepae9pg0db9vqaiVgFr0xfr=xfr=xc9adbaqaaeGacaGaaiaabeqaaeqabiWaaaGcbaGaem4saSKaeiikaGIaemOqai0aaSbaaSqaaiabdIha4bqabaGccqGGSaalcqWGcbGqdaWgaaWcbaGaemyEaKhabeaakiabcMcaPiabg2da9maaxababaGagiyBa0MaeiyyaeMaeiiEaGhaleaacqWGQbGAdaWgaaadbaGaemiEaGhabeaaliabcYcaSiabdQgaQnaaBaaameaacqWG5bqEaeqaaaWcbeaakiabdseaejabcIcaOiabdQgaQnaaBaaaleaacqWG4baEaeqaaOGaeiilaWIaemOAaO2aaSbaaSqaaiabdMha5bqabaGccqGGPaqkcqGGSaalaaa@4C56@

D(jx,jy)=max⁡{D(jx−1),jy)−pD(jx,jy−1)−pD(jx−1,jy−1)+f(jx,jy),
 MathType@MTEF@5@5@+=feaafiart1ev1aaatCvAUfKttLearuWrP9MDH5MBPbIqV92AaeXatLxBI9gBaebbnrfifHhDYfgasaacPC6xNi=xI8qiVKYPFjYdHaVhbbf9v8qqaqFr0xc9vqFj0dXdbba91qpepeI8k8fiI+fsY=rqGqVepae9pg0db9vqaiVgFr0xfr=xfr=xc9adbaqaaeGacaGaaiaabeqaaeqabiWaaaGcbaGaemiraqKaeiikaGIaemOAaO2aaSbaaSqaaiabdIha4bqabaGccqGGSaalcqWGQbGAdaWgaaWcbaGaemyEaKhabeaakiabcMcaPiabg2da9iGbc2gaTjabcggaHjabcIha4naaceqabaqbaeaabmqaaaqaaiabdseaejabcIcaOiabdQgaQnaaBaaaleaacqWG4baEaeqaaOGaeyOeI0IaeGymaeJaeiykaKIaeiilaWIaemOAaO2aaSbaaSqaaiabdMha5bqabaGccqGGPaqkcqGHsislcqWGWbaCaeaacqWGebarcqGGOaakcqWGQbGAdaWgaaWcbaGaemiEaGhabeaakiabcYcaSiabdQgaQnaaBaaaleaacqWG5bqEaeqaaOGaeyOeI0IaeGymaeJaeiykaKIaeyOeI0IaemiCaahabaGaemiraqKaeiikaGIaemOAaO2aaSbaaSqaaiabdIha4bqabaGccqGHsislcqaIXaqmcqGGSaalcqWGQbGAdaWgaaWcbaGaemyEaKhabeaakiabgkHiTiabigdaXiabcMcaPiabgUcaRiabdAgaMjabcIcaOiabdQgaQnaaBaaaleaacqWG4baEaeqaaOGaeiilaWIaemOAaO2aaSbaaSqaaiabdMha5bqabaGccqGGPaqkcqGGSaalaaaacaGL7baaaaa@7227@

where *f*(*j*_*x*_, *j*_*y*_) = 2·*exp*(-*γ*||bx,jx
 MathType@MTEF@5@5@+=feaafiart1ev1aaatCvAUfKttLearuWrP9MDH5MBPbIqV92AaeXatLxBI9gBaebbnrfifHhDYfgasaacPC6xNi=xH8viVGI8Gi=hEeeu0xXdbba9frFj0xb9qqpG0dXdb9aspeI8k8fiI+fsY=rqGqVepae9pg0db9vqaiVgFr0xfr=xfr=xc9adbaqaaeGacaGaaiaabeqaaeqabiWaaaGcbaacbeGae8Nyai2aaSbaaSqaaiabdIha4jabcYcaSiabdQgaQnaaBaaameaacqWG4baEaeqaaaWcbeaaaaa@32B9@ - by,jy
 MathType@MTEF@5@5@+=feaafiart1ev1aaatCvAUfKttLearuWrP9MDH5MBPbIqV92AaeXatLxBI9gBaebbnrfifHhDYfgasaacPC6xNi=xH8viVGI8Gi=hEeeu0xXdbba9frFj0xb9qqpG0dXdb9aspeI8k8fiI+fsY=rqGqVepae9pg0db9vqaiVgFr0xfr=xfr=xc9adbaqaaeGacaGaaiaabeqaaeqabiWaaaGcbaacbeGae8Nyai2aaSbaaSqaaiabdMha5jabcYcaSiabdQgaQnaaBaaameaacqWG5bqEaeqaaaWcbeaaaaa@32BD@||^2^) - 1, *D*(0, 0) = 0, *D*(*j*_*x*_, 0) = -*pj*_*x*_, *D*(0, *j*_*y*_) = -*pj*_*y*_, *γ *is the parameter of RBF kernel, and *p *is the gap penalty of the alignment. Note that the value of *f*(*j*_*x*_, *j*_*y*_) lies in the interval [-1, 1]. When bx,jx
 MathType@MTEF@5@5@+=feaafiart1ev1aaatCvAUfKttLearuWrP9MDH5MBPbIqV92AaeXatLxBI9gBaebbnrfifHhDYfgasaacPC6xNi=xH8viVGI8Gi=hEeeu0xXdbba9frFj0xb9qqpG0dXdb9aspeI8k8fiI+fsY=rqGqVepae9pg0db9vqaiVgFr0xfr=xfr=xc9adbaqaaeGacaGaaiaabeqaaeqabiWaaaGcbaacbeGae8Nyai2aaSbaaSqaaiabdIha4jabcYcaSiabdQgaQnaaBaaameaacqWG4baEaeqaaaWcbeaaaaa@32B9@ and by,jy
 MathType@MTEF@5@5@+=feaafiart1ev1aaatCvAUfKttLearuWrP9MDH5MBPbIqV92AaeXatLxBI9gBaebbnrfifHhDYfgasaacPC6xNi=xH8viVGI8Gi=hEeeu0xXdbba9frFj0xb9qqpG0dXdb9aspeI8k8fiI+fsY=rqGqVepae9pg0db9vqaiVgFr0xfr=xfr=xc9adbaqaaeGacaGaaiaabeqaaeqabiWaaaGcbaacbeGae8Nyai2aaSbaaSqaaiabdMha5jabcYcaSiabdQgaQnaaBaaameaacqWG5bqEaeqaaaWcbeaaaaa@32BD@ are similar, *f*(*j*_*x*_, *j*_*y*_) takes a positive value. Otherwise, *f*(*j*_*x*_, *j*_*y*_) takes a negative value.

The feature vector for representing a block is expressed by **b **= (*r*_1_, *r*_2_,...,*r*_20_), where *r*_1_, *r*_2_,...,*r*_20 _indicate the composition of 20 amino acids. Let *score*(*cTP*), *score*(*mTP*), *score*(*SP*), and *score*(*other*) be values of "discriminant" calculated for a protein sequence by gist-classify [33]. Our predictor selects max{*score*(*cTP*), *score*(*mTP*), *score*(*SP*), *score*(*other*)} and outputs the corresponding location. It is not guaranteed that the kernel matrix obtained from alignment scores is always valid (i.e., positive semi-definite). However, SVM training finished successfully in all cases of our computational experiments, which suggests that all the matrices used in the computational experiments can be treated as if it was positive semi-definite. Actually, in most cases, matrices produced by our method is not semi-definite since our method includes alignment. However, since absolute values of negative eigenvalues are small, SVM training can be executed successfully. If gap penalty and *γ *in Table 2 are not appropriately given, we cannot train SVM since absolute values of negative eigenvalues are too large.

### Data sets

The data sets used in this work were downloaded from TargetP [5] and WoLF PSORT [3]. TargetP data sets were collected from the SWISS-PROT database. Although TargetP data sets consist of plant and non-plant proteins, the mitochondrial proteins contain sequences from both plant and non-plant proteins since the number of plant mitochondrial proteins extracted from SWISS-PROT was too small to be used.

The redundancy reduced sets from which the training and test sets were built contain 141 cTP, 368 mTP, 269 SP, and 162 other (nuclear or cytosolic) sequences for plant and 371 mTP, 715 SP, and 1652 other (nuclear or cytosolic) sequences for non-plant as shown in Table 11.

**Table 11:** Number of sequences in each subcellular location of TargetP plant and non-plant data sets

Subcellular location	No. of sequences (plant)	No. of sequences (non-plant)
Chloroplast(cTP)	141	-
Mitochondrial(mTP)	368	371
Secretory(SP)	269	715
Nuclear+cytosolic(other)	162	1652

Total	940	2738

Matsuda et al. [22] checked the redundancy of this data set. They performed the prediction of subcellular location by using Smith-Waterman score, that is, the location of a sequence in the training data with a highest score is assigned to the corresponding query sequence in the test data. As a result, 75.7% and 84.0% of overall accuracies were obtained for plant and non-plant datasets respectively.

In order to perform a fivefold cross-validation test, each data set was partitioned into five subsets that have approximately equal sizes. Before partitioning, we randomly construct a permutation that consists of integers from 1 to 940 (or 2738) for plant (or non-plant). One subset is regarded as test data and the remaining four subsets as training data. This procedure is repeated five times so that each subset is used as test data once.

Plant protein data sets of WoLF PSORT are included in WoLF PSORT package version 0.2 that can be downloaded from [30]. By removing dual localization proteins, we obtained 2391 proteins in which 11 kinds of subcellular location exist. There was 35 proteins which have dual locations. Details are shown in Table 3.

### Accuracy measures

In order to implement SVM, we used the software GIST [33]. We evaluated the prediction performance of our method by calculating sensitivity, specificity, Matthew's correlation coefficient (MCC) [34], and overall accuracy for each subcellular location. The definitions of these measures are as follows:

Sensitivity(l)=tp(l)tp(l)+fn(l),Specificity(l)=tp(l)tp(l)+fp(l),
 MathType@MTEF@5@5@+=feaafiart1ev1aaatCvAUfKttLearuWrP9MDH5MBPbIqV92AaeXatLxBI9gBaebbnrfifHhDYfgasaacPC6xNi=xI8qiVKYPFjYdHaVhbbf9v8qqaqFr0xc9vqFj0dXdbba91qpepeI8k8fiI+fsY=rqGqVepae9pg0db9vqaiVgFr0xfr=xfr=xc9adbaqaaeGacaGaaiaabeqaaeqabiWaaaGcbaqbaeqabeGaaaqaaiabdofatjabdwgaLjabd6gaUjabdohaZjabdMgaPjabdsha0jabdMgaPjabdAha2jabdMgaPjabdsha0jabdMha5jabcIcaOiabdYgaSjabcMcaPiabg2da9KqbaoaalaaabaGaemiDaqNaemiCaaNaeiikaGIaemiBaWMaeiykaKcabaGaemiDaqNaemiCaaNaeiikaGIaemiBaWMaeiykaKIaey4kaSIaemOzayMaemOBa4MaeiikaGIaemiBaWMaeiykaKcaaOGaeiilaWcabaGaem4uamLaemiCaaNaemyzauMaem4yamMaemyAaKMaemOzayMaemyAaKMaem4yamMaemyAaKMaemiDaqNaemyEaKNaeiikaGIaemiBaWMaeiykaKIaeyypa0tcfa4aaSaaaeaacqWG0baDcqWGWbaCcqGGOaakcqWGSbaBcqGGPaqkaeaacqWG0baDcqWGWbaCcqGGOaakcqWGSbaBcqGGPaqkcqGHRaWkcqWGMbGzcqWGWbaCcqGGOaakcqWGSbaBcqGGPaqkaaGccqGGSaalaaaaaa@7A9A@

MCC(l)=tp(l)⋅tn(l)−fp(l)⋅fn(l)(tp(l)+fn(l))(tp(l)+fp(l))(tn(l)+fp(l))(tn(l)+fn(l)),
 MathType@MTEF@5@5@+=feaafiart1ev1aaatCvAUfKttLearuWrP9MDH5MBPbIqV92AaeXatLxBI9gBaebbnrfifHhDYfgasaacPC6xNi=xI8qiVKYPFjYdHaVhbbf9v8qqaqFr0xc9vqFj0dXdbba91qpepeI8k8fiI+fsY=rqGqVepae9pg0db9vqaiVgFr0xfr=xfr=xc9adbaqaaeGacaGaaiaabeqaaeqabiWaaaGcbaGaemyta0Kaem4qamKaem4qamKaeiikaGIaemiBaWMaeiykaKIaeyypa0tcfa4aaSaaaeaacqWG0baDcqWGWbaCcqGGOaakcqWGSbaBcqGGPaqkcqGHflY1cqWG0baDcqWGUbGBcqGGOaakcqWGSbaBcqGGPaqkcqGHsislcqWGMbGzcqWGWbaCcqGGOaakcqWGSbaBcqGGPaqkcqGHflY1cqWGMbGzcqWGUbGBcqGGOaakcqWGSbaBcqGGPaqkaeaadaGcaaqaaiabcIcaOiabdsha0jabdchaWjabcIcaOiabdYgaSjabcMcaPiabgUcaRiabdAgaMjabd6gaUjabcIcaOiabdYgaSjabcMcaPiabcMcaPiabcIcaOiabdsha0jabdchaWjabcIcaOiabdYgaSjabcMcaPiabgUcaRiabdAgaMjabdchaWjabcIcaOiabdYgaSjabcMcaPiabcMcaPiabcIcaOiabdsha0jabd6gaUjabcIcaOiabdYgaSjabcMcaPiabgUcaRiabdAgaMjabdchaWjabcIcaOiabdYgaSjabcMcaPiabcMcaPiabcIcaOiabdsha0jabd6gaUjabcIcaOiabdYgaSjabcMcaPiabgUcaRiabdAgaMjabd6gaUjabcIcaOiabdYgaSjabcMcaPiabcMcaPaqabaaaaOGaeiilaWcaaa@8B40@

Overall accuracy=1m∑l=1ktp(l),
 MathType@MTEF@5@5@+=feaafiart1ev1aaatCvAUfKttLearuWrP9MDH5MBPbIqV92AaeXatLxBI9gBaebbnrfifHhDYfgasaacPC6xNi=xI8qiVKYPFjYdHaVhbbf9v8qqaqFr0xc9vqFj0dXdbba91qpepeI8k8fiI+fsY=rqGqVepae9pg0db9vqaiVgFr0xfr=xfr=xc9adbaqaaeGacaGaaiaabeqaaeqabiWaaaGcbaGaem4ta8KaemODayNaemyzauMaemOCaiNaemyyaeMaemiBaWMaemiBaWMaeeiiaaIaemyyaeMaem4yamMaem4yamMaemyDauNaemOCaiNaemyyaeMaem4yamMaemyEaKNaeyypa0tcfa4aaSaaaeaacqaIXaqmaeaacqWGTbqBaaGcdaaeWbqaaiabdsha0jabdchaWjabcIcaOiabdYgaSjabcMcaPaWcbaGaemiBaWMaeyypa0JaeGymaedabaGaem4AaSganiabggHiLdGccqGGSaalaaa@52FF@

where *m *is the total number of protein sequences and *k *is the number of subcellular locations. *tp*(*l*) is the number of correctly predicted sequences belonging to location *l *(true positive). *tn*(*l*) is the number of correctly predicted sequences that do not belong to location *l *(true negative). *fp*(*l*) is the number of overpredicted sequences in location *l *(false positive). *fn*(*l*) is the number of underpredicted sequences in location *l *(false negative).

## Availability and requirements

Project name: SLPFA;

Project home page: ;

Operating system(s): Platform independent;

Programming language: none;

License: no restriction;

Any restrictions to use by non-academics: no restriction.

## Authors' contributions

TT designed the detailed methods and algorithms, managed the computer experiment and constructed the web-based system. TA proposed the basic idea of the methods and supervised this work. Both authors read and approved the final manuscript.
